# Progress in Optimization of *Agrobacterium-*Mediated Transformation in Sorghum (*Sorghum bicolor*)

**DOI:** 10.3390/ijms19102983

**Published:** 2018-09-29

**Authors:** Rana Imtiaz Ahmed, Anming Ding, Minmin Xie, Yingzhen Kong

**Affiliations:** 1 Key Laboratory for Tobacco Gene Resources, Tobacco Research Institute, Chinese Academy of Agricultural Sciences, Qingdao 266101, China; imtiazheaven@yahoo.com (R.I.A.); dinganming@caas.cn (A.D.); xieminmin@caas.cn (M.X.); 2 Graduate School of Chinese Academy of Agricultural Science, Beijing 100081, China

**Keywords:** T-DNA, antibiotics, phenolic compounds, immature embryos, tissue culture

## Abstract

This review archives the achievements made in the last two decades and presents a brief outline of some significant factors influencing the *Agrobacterium*-mediated transformation of *Sorghum bicolor*. Recently, progress in successful transformation has been made for this particular monocot crop through direct DNA delivery method and indirect method via *Agrobacterium.* However, lower transformation rate still proved to be a bottleneck in genetic modification of sorghum. An efficient *Agrobacterium* transformation system could be attained by optimizing the preliminary assays, comprising of explant source, growth media, antibiotics, *Agrobacterium* strains and agro-infection response of callus. The selection of competent strains for genetic transformation is also one of the key factors of consideration. Successful transformation is highly dependent on genome configuration of selected cultivar, where non-tannin genotype proved the best suited. Immature embryos from the field source have higher inherent adaptation chances than that of the greenhouse source. A higher concentration of *Agrobacterium* may damage the explant source. Utilization of anti-necrotic treatments and optimized tissue culture timeframe are the adequate strategies to lower down the effect of phenolic compounds. Appropriate selection of culture media vessels at different stages of tissue culture may also assist in a constructive manner. In conclusion, some aspects such as culture environment with medium composition, explant sources, and genotypes play an indispensable role in successful *Agrobacterium*-mediated sorghum transformation system.

## 1. Introduction

Sorghum (*Sorghum bicolor*) is ranked fifth among cereal crops, with well understood agronomic practices and uses that make it considered as the food for all [1]. Owing to its miscellaneous efficacy even in harsh climates, more than 500 million lives in Africa and Asia are directly or indirectly dependent on this crop [2]. It is considered as the first C4 high cellulosic biofuel feedstock crop and is a model monocot [3], belonging to the Andropogoneae tribe with chromosome number (2*n* = 20). Its genome contains 750 Mb of DNA, which is 3–4 times smaller than maize but larger than that of rice (430 Mb), making sorghum an attractive species to study in the *Poaceae* family [4].

To meet the inexorable global population pressure by 2030, drought-resistant sorghum is the best-suited cereal crop for peripheral land cultivation, wherein adverse growing conditions, e.g., water shortage and logging, alkalinity, salinity, and other constraints, exist. It is becoming widely cultivated sugar crop with tremendous potential for bioenergy and ethanol production per unit area of land, which is a splendid replacement in energy deficit industries, especially for underdeveloped countries. For gluten sensitive enteropathic sufferers, highly enriched antioxidant sorghum is a striking grain replacement. Despite its unique features, there is still space available to improve the considerable traits in a modified manner for subsequent exploitation [5].

To intensify the plant development, genetic transformation proved to be a powerful tool by gene induction, modulation and expression [6]. For example, Cook et al. [7] demonstrated the role of two alkylresorcinol synthases in the production of allelopathic molecule sorgoleone by reducing its expression in sorghum. However, sorghum has been classified as one of the most challenging plant species to perform tissue culture and genetic transformation [8]. An effectual gene transmission basically depends upon improved tissue culture techniques and sophisticated DNA delivery system. Significant achievement has been made in the above-mentioned areas [9,10,11,12,13,14,15,16,17]. *Agrobacterium* mediation and micro particle-bombardment transformation are two main approaches that have been exploited for the accomplishment of transgenic sorghum. In 1993, the first successful transgenic sorghum via particle bombardment technique with 0.28% transformation rate was attained [18]. After that, Zhao et al. [19] reported the *Agrobacterium*-mediated transgenic sorghum with an improved rate of 2.12%. Since the first successful *Agrobacterium-*mediated transformation, protocols utilizing explant tissues and several compatible sorghum genotypes have been reported by researchers [10,20,21,22,23,24,25]. Similar to monocotyledonous crops, in vitro culture system for sorghum is primarily somatic embryogenesis based [26,27,28,29,30]. Even though for stable sorghum transformation recovery both direct and indirect gene transfers have been adopted, indirect method, i.e., *Agrobacterium*-mediated transformation system, is preferred by most researchers [31]. The transformants by *Agrobacterium*-mediation have the capacity to produce lower copy insertions and have a higher rate of co-expression of the non-selected transgenic cassette [19,32]. An improved grain lysine content gene such as *HT-*12 which is a form of hordothionin [33] and a gene *cry1Ac* for insect resistance have been successfully incorporated in sorghum [34]. Although researchers have been succeeded in widely adopted agronomic transformed sorghum, the optimized transformation system is still to be achieved [30].

One of the important features of sorghum is the ability to outcross with its weedy relatives such as Johnson grass (*Sorghum halepense*). The utilization of non-herbicide resistant marker gene is a positive approach in an efficient sorghum transformation system. Sorghum is deliberately known as the richest source of phenolic compounds along with different flavonoids and condensed tannins by varying levels [35]. It is not necessary that every genotype has tannin grain. According to Hahn et al. [36], the phenolic acids are mainly in bounded form with ferulic acid being dominant, ranging 24–47%. The hasty production of phenolic metabolites during the inoculation with *Agrobecterium tumefaciens* and in vitro culturing is a major constraint that drastically lowers the efficiency rate. To alleviate the phenolic effect, utilization of antioxidants and absorbing agents can be helpful in culture medium.

### 1.1. Factors Influencing the Sorghum Transformation

Based on numerous studies, it is revealed that *Agrobacterium*-mediated transformation of sorghum is the most challenging activity due to many factors. Prior to commencing the stable transformation trials, it is highly recommended to evaluate the basic necessary components and conditions. Along with the selection of suitable genotypes, a source of explant, *Agrobacterium* strains with comparative plasmid construction and optimized media culture, should be considered.

### 1.2. Genotype

Among monocotyledonous species that have been transformed up until now, noticeably *Oryza sativa* is the most genotype independent, while, in sorghum, transformation efficiency deviates from cultivar to cultivar. It is difficult to conclude whether this genotypic dependency is due to T-DNA delivery or in vitro tissue culture response. Non-tannin genotypes are best suited for transformation, supporting the findings of Casas et al. [37], who failed to transform the high tannin genotype (IS4225) via microprojectile bombardment. Immature embryos sourced from different genotypes have been utilized by researchers with varying transformation rate. These genotypes included cultivars and hybrids of sorghum. A renowned public cultivar P898012 having pre- and post-flowering drought resistance [37] is widely used in *Agrobacterium-*mediated transformation system. Zhao et al. [19] gave the first breakthrough by achieving the transformation of P898012 and PH1391. Pioneer 8505, a commercial hybrid, and an inbred line C401 were utilized by Gao et al. [20]. Howe et al. [22] utilized immature embryos from Tx430 and C2-97 in their experiments and achieved up to 4.5% transformation efficiency. Sensako 85/1191 red cultivar of sorghum was used by Nguyen et al. [25]. Genotypes P898012 and RTX 430 were utilized by Kumar et al. [38] who found expected results, although the success rate was very low. Do et al. [39] worked on regeneration tests of five sorghum genotypes, viz. P898012, TBx623, Tx2737, Tx430, and Wheatland, and found P898012 as the most suitable for the transformation by achieving the efficiency of transformation up to 14% in the experiments. Tx430, a non-tannin sorghum variety, was used to study the effects of CuSO_4_ and BAP with varying concentrations in resting and selection media during transformation by Wu et al. [40]. Jeoung et al. [13] utilized Tx430, C401, C025, and Wheatland in *Agrobacterium* transformation experiments with low transformation efficiency. Panday et al. [41] utilized shoot apices of genotypes BTx623 and M35-1 in their research work for the study of transient gus expression.

### 1.3. Sources of Explant

**Figure 1:**
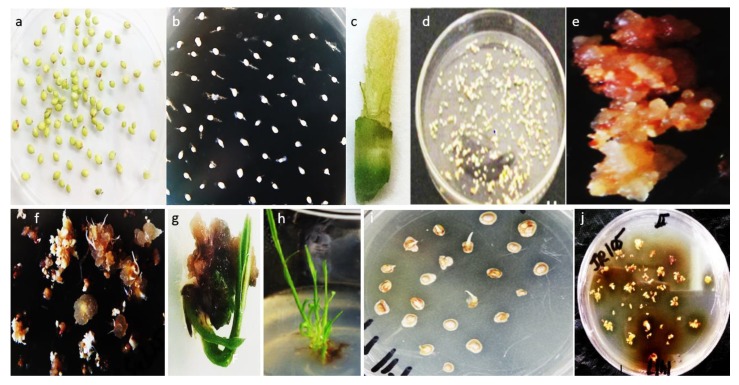
Different stages of sorghum transformation: (**a**) immature Embyos after 14 days of pollination; (**b**) explant source-immature embryos; (**c**,**d**) explant source-immature inflorescence; (**e**,**f**) callus induction from immature inflorescence; (**g**) regeneration of shoo;t (**h**) regeneration of root; (**i**) excessive *Agrobacterium* growth around explant seize the callus induction; and (**j**) production of phenolic compounds resulted in ultimate death of callus.

Initially, Hiei et al. [42] reported mature seeds as the best explant source in *japonica* rice for *Agrobacterium*-mediated transformation due to its active cell division. Later, it was protracted to other monocotyledonous crops, such as wheat (*Triticum aestivum*) [43,44], maize (*Zea mays*) [45,46], barley (*Hordeum vulgare*) [47] and sorghum [19]. Freshly isolated immature embryos showed more compatibility toward agro-infection transformation [13,20,22,39,40]. Immature embryos, as shown in Figure 1a,b, taken after 12–16 days of pollination gave good response to callus induction. An appropriate in vitro culture system can be obtained by pre-culturing the immature embryos prior to the agro-infection. Similar to other monocotyledonous crops, it was observed that cell culture for short span of time reduced T-DNA delivery [48]. Desiccation treatment to immature embryos before *Agrobacterium* infection resulted in the stable transformation in some monocot crops [43], while that phenomenon was not observed in the case of sorghum; only fresh immature embryos explants were found to be competent for transformation. Instead, Nguyen et al. [25] reported one-day cold pre-treatment at 4 °C on immature seeds prior to the excision of immature embryos contributed positive effect on callus formation and survival along with reduced need for regular subculture. Besides the active participation of immature embryos in the in vitro callus regeneration system, the availability is limited and time specific, resulting in barricade to the smooth transformation process. To overcome this hurdle, a few other resources of explant such as immature inflorescence, as shown in Figure 1c, via microprojectile bombardment [49], shoot apices [41], piercing to mature embryo seeds [50] and mature seeds for in vitro culture [28], have been utilized with low transformation rates. The explants from field source have a consequential effect on transformation frequency in relation to greenhouse stock [19]. The published reports concluded that immature embryos and immature inflorescence were the best explant sources for callus induction while piercing of mature embryo seed transformation process escaped the callus induction step resulting in the less required timeframe for transformation and labor cost.

### 1.4. Agrobacterium Strains and Vectors

*Agrobacterium* is a Gram-negative soil bacterium that utilizes horizontal gene transfer to produce a tumor in plants. Inoculation with *Agrobacterium* often results in necrosis [51,52]. The virulence of the selected strain is possibly the most critical concern in callus response to agro-infection [53]. In rice, after the successful utilization of combinations of either super-virulent strain (EHA101) harboring standard binary vectors or a regular strain (LBA4404) retaining a super-binary vector with extra copy of *vir*B, *vir*C, and *vir*G genes, many researchers adopted similar combinations in monocots, e.g., rice [54], maize [45,55], barley [47] and sorghum [19]. A combination of *Agrobacterium* strain coupled with suitable vector effect the transformation rate. Although it is not necessary that these combinations will always give positive results, there may be variation in transformation efficiency [56]. *Agrobacterium tumefaciens* LBA4404 with standard binary vector implies an efficient transformation system in maize with optimized co-culture and regeneration medium [57]. By using *Agrobacterium* strain AGL1 with modified protocol, Wu et al. (2014) [40] reported 33% increase in the transformation efficiency. NTL4, EHA101, and GV3101, important *Agrobacterium* strains that have given comprehensive results with varying degrees of transformation efficiency, have been employed by many researchers in their experiments [20,22,39] (for more references, see Table 1).

### 1.5. Agrobacterium Concentration

An optimized concentration of *Agrobacterium* is recommended. In rice, Hiei et al. [42] observed the transformation when *Agrobacterium* density range was 1.0 × 10^6^–1.0 × 10^10^ cfu/mL. Ishida et al. [45] reported the same *Agrobacterium* density in maize by achieving the stable transformation events. Zhao et al. [46] testified the role of the N6-based medium in maize for the optimization of *Agrobacterium* densities. The concentration may vary according to genotype. In some cultivars of sorghum, 1 × 10^9^ cfu/mL gave lesser transformation rate as compared to 0.5 × 10^9^ cfu/mL. The range 0.5–0.7 × 10^9^ cfu/mL may be suitable for sorghum transformation. Through various experiments, it was justified that higher *Agrobacterium* density reduced the callus initiation frequency, as shown in Figure 1i, while transient gus activity increased [19]. The same phenomenon of correlation of higher *Agrobacterium* densities was studied in wheat [44]. 

### 1.6. Marker Selection and Reporter Genes

A balanced transformation system comprises the choice of visual and selectable marker genes for rapid and efficient identifying of transgenic cells from the non-transgenic cell lineage. Selectable marker genes normally depend on providing resistance to antibiotics, such as aminoglycoside kanamycin, with their various derivatives [69], hygromycin phosphotransferase [70] and the tolerance towards herbicidal agents glufosinate [71] and glyphosate [72]. Ecological and consumer concerns regarding the expected consequences of antibiotic and herbicidal resistant genes, led the new selection approach by which transgenic cells are provided a metabolic advantage over non-transgenic cells [73]. *Phosphomannose isomerase* (PMI), the positive selectable marker gene [74], has been shown to be rather a competitive selection system for the identification of transgenic plants [55,75]. In sorghum transformation, reporter genes encoding β-glucuronidase (GUS) or green fluorescent protein (GFP) have been exploited extensively [76]. Both are effective for differentiation of transgenic cells [13] (for more references, see Table 1).

### 1.7. In Vitro Culture Media Composition

To obtain the stable transformation, the media may be manipulated towards optimization by improving the capability of plant target cells to T-DNA delivery, and an efficient post infection plant cell recovery. Different chemicals are utilized in a systemic manner to achieve the stable transformation. The whole transformation process can be split into five major steps, which can be further divided: inoculation, co-cultivation, resting period, callus induction medium and regeneration process.

A well-balanced composition of culture media allows the smooth genetic transformation. Zhao et al. [19] worked on a balanced and comprehensive growth media for *Agrobacterium*-mediated sorghum transformation, by obtaining 131 stable transformed events from 6175 agro-infected immature embryos. The protocol was optimized with the addition of vitamin stock (nicotinic acid, pyridoxine HCl and thiamin HCl) in every stage of growth media from inoculation to rooting phase. To lower the effect of necrosis by the production of phenolic compounds, coconut water was used in resting media onward. For initiation of cell wall, zeatin in regeneration and naphthalene acetic acid (NAA) for root induction was utilized in the culture media. Gao et al. [20] reported 3.3% transformation rate by addition of phytagel to co-cultivation, callus induction and rooting media and kinetin to the regeneration medium instead of zeatin along with vitamin stock solution of varying concentrations. Nguyen et al. [25] optimized the callus induction medium by adding activated charcoal (AC), enzymatic casein hydrolysate (CH) and 2,4-dichlorophenoxyacetic acid (2,4-D), whereas growth nutrients were supplied by MS salts [77], B5 vitamins [78] and sucrose. The medium was solidified by GibcoBRL 0.8% agar at pH 5.8. Furthermore, modified vessels such as vented petri dishes also have an effect on the transformation efficiency, especially by increasing discharge rate of gases, e.g., ethylene produced during rapid growth of callus and regeneration phases by minimizing the adverse effect of high accumulation levels of such exudates [25]. Kumar et al. [38] modified the callus induction medium by proline along with asparagine, while improved B5 organics [37] were utilized in regeneration phase. Winans et al. [79] depicted better results in the transformation efficiency by refined *Agrobacterium*-induction medium with lowering the phosphate and maintaining the acidic pH of the AB minimal medium. Wu et al. [40] was able to get enhanced transformation efficiency by fast growing, high quality and re-generable transgenic callus by addition of copper sulfate (CuSO_4_) and 6-benzylaminopurine (BAP) in the resting phase [19] (for more references, see Table 2). Do et al. [39] reported 14% transformation efficiency by reducing the tissue culture period up to seven weeks.

### 1.8. Osmotic Treatment

Sucrose and glucose have been extensively used in media preparation for monocot transformation via *Agrobacterium* and especially in biolistic-mediated transformation for osmotic effect. It was supposed that osmotic effect by these compounds had a positive role on stable transformation, however, the phenomenon is not yet elucidated. This has been exemplified by a number of researchers in their experiments by the inclusion of these compounds in the media for monocot crops such as maize and rice [46]. In sorghum, Zhao et al. [19] utilized sucrose for plasmolysis in all above-mentioned stages while glucose was used only during inoculation and co-cultivation period at the concentrations of 36 g/L and 10 g/L, respectively. Gao et al. [20] reported stable transformation by the addition of sucrose during inoculation with 68.5 g/L, co-cultivation with 20 g/L and callus initiation with 30 g/L, whereas glucose in the concentrations of 36 g/L and 10 g/L, respectively, was used during inoculation and co-cultivation.

### 1.9. Antioxidants

For limiting the oxidative effect, ascorbic acid has been utilized with varying concentration in monocot crops, especially in sorghum for genetic transformation. Ascorbic acid helps in the survival of the target explant after the inoculation of *Agrobacterium* by serving as an antioxidant. Enríquez-Obregón et al. [80] documented stably transformed callus of sugarcane by manipulating the media with silver nitrate, ascorbic acid and cysteine at the concentration of 2 mg/L, 15 mg/L and 40 mg/L, respectively. Zhao et al. [19] reported stable transformation of sorghum by the inclusion of ascorbic acid at the rate of 10 mg/L in the media while several other researchers followed the same media composition [39,40]. Silver nitrate inhibits the *Agrobacterium* growth without indicating any effect on T-DNA delivery. It is evident that cysteine significantly enhances the transient β-glucuronidase (GUS) expression in target cells in maize [57], however, followed by co-culture, it initiates cell necrosis by browning in soybean [81,82].

### 1.10. Antibiotics

Rafat et al. [83] proposed that Minimum Inhibitory Concentration (MIC) for the antibiotic should be evaluated in pre-transformation experiments. To suppress or eliminate the excessive growth of *Agrobacterium* following the co-culture, antibiotics such as carbenicillin, cefotaxime, and timentin have been used extensively for the *Agrobacterium*-mediated transformation of monocot crops [44,84]. Ishida et al. [45] reported cefotaxime at a concentration of 250 mg/L had harmful effects on maize *Agrobacterium* transformation. It had a detrimental effect on callus formation in callus induction medium and comparatively with carbenicillin giving three times less transformation efficiency [46]. Several reports indicated carbenicillin as an extensively used antibiotic in *Agrobacterium*-mediated transformation experiments for monocot crops such as wheat and maize [43,44,58]. It has been used at the concentration of 100 mg/L in stable transformation events of sorghum [19,20]. Kumar et al. [38] and Do et al. [39] reported cefotaxime at concentrations of 100 mg/L and 300 mg/L, respectively.

### 1.11. Phenolic Compounds

During the in vitro culture of the sorghum, the production of phenolic compound is considered to be the main constraint in efficient *Agrobacterium* transformation system by any explant material. This might have a negative effect on tissue growth, quality, and transformation rate, as shown in Figure 1h. Earlier, this problem was dispensed by addition of polyvinylpolypyrrolidone (PVPP) and providing short span subculture [19,20]. However, there are certain limitations as frequent subculture increases the labor cost and PVP can interact with an efficient concentration of growth regulators affecting the in vitro tissue growth. To deal with this issue, Elkonin and Pakhomova [12] worked on various media composition for the elevation of the negative impact of the phenolic compounds. One of these compositions, designated M11, gave substantial results by lowering the phenolic compounds in the in vitro culture and producinb quality events [30]. Nguyen et al. [25] studied the role of activated charcoal (AC) in the callus induction medium and pre-treatment of immature embryos at 4 °C for one day. Activated charcoal inhibited the production of the black pigments along with restriction of the tissue culture growth from immature embryo while cold treatment not only delayed the production of the phenolic compounds but also gave the vigorous growth to the tissues. These results are also supported the study of Kowalsky and van Staden [85] for the cold treatments of woody plants cultures for phenolic compounds. The addition of activated charcoal (AC) is useful, especially in anther cultures and for the formation and transmission of woody plant cultures, but not for sorghum.

### 1.12. Temperature

Every stage of in vitro culture needs to be evaluated with optimal temperature. The effect of temperature on T-DNA delivery during co-culture was first investigated in dicot plant species. Dillen et al. [86] reported 22.8 °C as an optimal temperature for T-DNA delivery in model plant, tobacco. Salas et al. [87] depicted the highest number of transformed events in tobacco by utilizing 25.8 °C during co-culture, even though 19.8 °C was found optimal for T-DNA delivery. These results clearly show the contradiction in temperature optimization for T-DNA delivery for stable transformation with a given species and explant. In monocots, the temperature during co-culture fluctuates between 24 and 25.8 °C, with the exception of 28.8 °C being found optimal in some cases [88,89,90]. Kondo et al. [91] observed highest transient gus expression at 22.8 °C with garlic calli. Frame et al. [57] demonstrated 20.8 °C achieves higher transformation frequency in maize by using a standard binary vector as compared to 23.8 °C. Gurel et al. [6] discovered that heat treatment of sorghum immature embryos of genotype P898012 at 43 °C for 3 min followed by cooling at 25 °C prior to inoculation significantly improved GFP-expressing calli and transformation frequency up to 8.3%. Nguyen et al. [25] showed that a pretreatment of immature embryos at 4 °C for one day had a positive effect on callus induction. Adkins et al. and Kozai and Smith [92,93] reported the role of culture vessels and their effects in rice and wheat for callus formation and plant regeneration.

### 1.13. Conclusion and Future Directions

**Figure 2:**
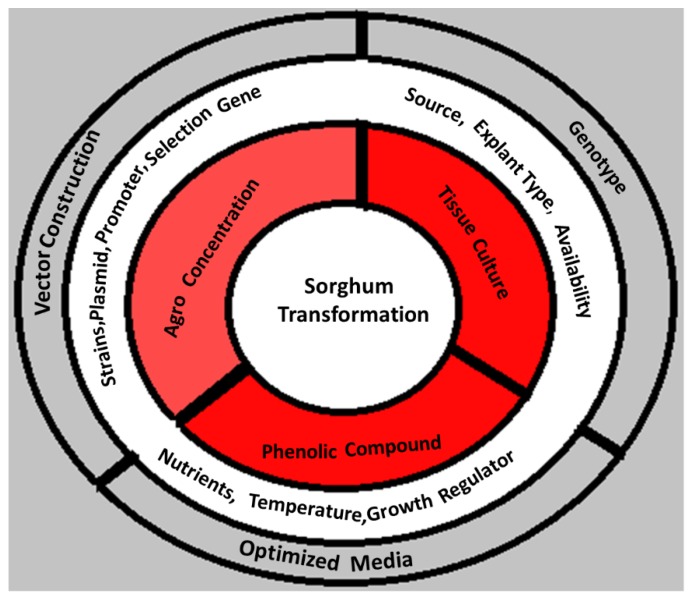
Diagrammatic representation of the whole Sorghum transformation process. The outer ring illustrates the components necessary for *Agrobacterium* transformation, the second ring focuses on the important factors affecting the transformation process and the inner third ring describes the problems with different color intensities occurring during the sorghum transformation.

Since the initial breakthrough in the *Agrobacterium*-mediated transformation of sorghum in early 2000, progress has been made in media optimization and vector–explant selection. Although researchers have succeeded to make a breach in one of the most recalcitrant crops, the transformation rate is still not high enough and needs improvement, e.g., the use of readily available explants, simple media optimization rather complex optimization, lower in vitro culture length, the possible solution of deadly phenolic compounds and wide range of compatible genotypes. The complexity of this process is illustrated in Figure 2. As mentioned above, the utilization of immature embryo in sorghum transformation is highly time specific element so there is need to expand the compatible explant spectrum, which is basically the non-immature embryo-based system, by manipulating them to hot and cold treatments, anti-phenolic treatments and desiccation [94]. Along with these parameters, the understanding of vector manipulation is also important, i.e., the gene of interest with suitable selection marker gene, T-DNA size, *vir*G genes which enhances the integration of T-DNA, and replication-associated protein (RepA) that stimulates the cell division [95] can be helpful to increase the stable transformation. Moreover, the utilization of proper or specialized ventilated vessels might play an important role, as Ezeogu et al. [96] reported lower gene transformation due to the accumulation of excessive ethylene production in vessels after the agro-infection. Furthermore, only genotypes of sorghum that were already considered model genotypes for particle bombardment transformation have been subjected to *Agrobacterium*-transformation, therefore, the circle of *Agrobacterium*-mediated transformation should be extended to other elite genotypes.

The endosperm of sorghum grain store major proteins, e.g., prolamin known as kafirin. Its digestibility is limited because its much-defined pattern eventually influences the nutritional quality of the grain [97,98]. Hence, prolamins digestibility modulation could lead to enhancing the nutritional value in the long run. Oria et al. [99] reported a highly digestible, lysine supplemented sorghum mutant. Other miscellaneous issues related to biofuel industries such as variation in Brix percentage, juice sucrose concentration and total stalk sugar yield along with pre- and post-flowering drought resistance with other agronomic characters can be addressed by the help of better understanding of molecular breeding and biotechnology tools. Nevertheless, researchers have not succeeded in developing an ideal transformation system for this tough and tedious crop, however the progress that has been made is a spot light towards unveiling the ultimate success.

## Figures and Tables

**Table 1:** Transformation of Sorghum via *Agrobacterium* and Biolistic Approach.

Explants	*Agrobacterium* Strain	Vectors	Selection Marker	Promoter Used for Selection Marker	Reporter Gene	Promoter Used for Reporter Gene	Transgene Detection/Expression	Transformation Efficiency	Key Points/Studied Parameters	References
Immature Embryo	LBA4404	pSB1 pSB11	*bar*	Ubi1	*gus*	Ubi1	Southern Blot	2.1%	Study on protocol optimization and source of genotype.	Zhao et al. [19]
Immature Embryo	EHA105 EHA101 AGL1	pPZP200	*hbt*	Ubi1, CaMV35S	*gfp*, *gus*	Hbt chimeric promoter with CaMV35S enhancer	PCR	Only a few events	Compare gfp and gus reporter genes by direct and indirect gene transfer methods.	Jeoung et al. [13]
Immature Embryo	LBA4404	pTOK233	*hpt*	CaMV35S	*gus*	CaMV35S	PCR, Southern Blot	1.7–3.5%	Media optimization by antioxidants and study transient *gus* expression.	Carvelho et al. [10]
Immature Embryo	EHA101	pPZP201	*pmi*	Ubi1	*gfp*	Ub1	Southern Blot, Western Blot, CPR Assay, PCR	2.8–3.3%	Study *pmi* gene as a selectable marker on mannose selective agent.	Gao et al. [20]
Immature Embryo	NTL4	pPZP212	*npt II*	Ubi1	*gus*	Ubi1	Southern blot	0.3–4.5%	Utilized standard binary vector with *npt II* gene as a selectable marker.	Howe et al. [22]
Immature Embryo	LBA4404	pCAMBIA1301	*hpt*	CaMV35S	*gus*	CaMV35S	Southern Blot PCR	5%	Apply cold-pre-treatment on explant, increase callus induction and reduced phenols.	Nguyen et al. [25]
Immature Embryo	EHA101 LBA4404	pPZP201	*pmi*	Ubi1	*gfp*	Ubi1	PCR, Western Blot	8.3%	Treatment to immature embryos at 43°C, with various time frames.	Gurel et al. [6]
Immature Inflorescence	EHA105	pKUB	*hpt*	Ubi1	*gus*	CaMV35S	RT-PCR, Southern Blot, Western Blot	1.9%	Incorporation of insecticidal *cry1Ab* gene.	Zhang et al. [58]
Immature Embryo	EHA105, EHA101, LBA4404	pMKURF2, pCAMBIAG11 pCAMBIARC7	*hpt*	Ubi1	*gus*	Ubi1	Western blot	1.6–2.7%	Study of pathogen resistance, chitinase.	Arulselvi et al. [59]
Mature Embryo, Young Seedling, Immature Inflorescence	LBA4404	pKU352NA	*hpt*	Ubi1	*SgfpS65T,*	Ubi1	Inverse PCR	4.28%	Improved *gfp* and Ac-Ds system.	Jambagi et al. [23]
Immature Embryo	NTL4	pCAMBIA1305.2 pCAM-UBIgus	*hpt*	Ubi1	*gus*	Ubi1	PCR, Southern Blot	2.4%	Study the effect of *L*-cystine and inclusion of additional binary vector.	Kumar et al. [38]
Shoot Apices	EHA105	pCAMBIA1305			*gus*	CaMV35S	Histochemical *gus* assay	Few transient event	Effect of cysteine on *gus* activity.	Pandey et al. [41]
Shoot Apices	LBA4404	pCAMBIA1305.1	*hpt*	CaMV35S	*gus*	CaMV35S	PCR, Southern Blot	1.2–3.9%	Insect resistance, *cry1gene*	Ignacimuthu and Premkumar [60]
Immature Embryo	LBA4404 AGL1	pSB1 pSB11	*pmi*	Ubi1	*DsRed*	Ubi1	QPCR	10% by LBA4404 & 33% by AGL1	Effect of CuSO_4_ and BAP in resting and selection media.	Wu et al. [40]
Immature Embryo	AGL1 EHA101 GV3101	pZY102, pFGC5941 pFGC161	*bar*	MAS, ZmUbi1	*gus*	CaMV35S	PCR Southern blot	14%	Study standard binary vector and *bar* gene as a selectable marker.	Do et al. [39]
Mature Embryo	EHA105	pCAMBIA1305.1 pCUbi1390	*hph*	Ubi1	*gfp*	CaMV35S	PCR, Western Blot	4%	Piercing the mature seeds excluding the tissue culture process	Li et al. [50]
Immature Embryo	EHA105	pHP78891		Ubi1	*gfp*	Ubi1	PCR, Southern Blot	6.2%	Study of morphogenic regulator, BABY BOOM, WUSCHEL2	Mookkan et al. [61]
**Gene Transformation Through Biolistic Approach**										
Immature Embryo	biolistic	pBCI pNGI	*hpt*	Adh1	*gus*	Adh1	Gus Assay, RNA gel blot analysis	Observe transient events	Study of hygromycine and kanamycine resistance gene.	Hagio et al. 1991 [62]
Immature Embryo	biolistic	pPHP620 pPHP687	*bar*	D-CaMV35S	*gus*	D- CaMV35S	Southern Blot Gus assay	0.08%	Introduction of the bar gene.	Casas et al. 1993 [37]
Immature Embryo	biolistic		*bar*				Southern Blot, Western Blot, PCR	0.09%	Introduction of chitinase *G11* gene.	Zhu et al. [8]
Immature Embryo, Leaf Segment	biolistic	pAHC20	*bar*	Ubi1, Actin, CaMV35S	*gfp*	Ubi1 Actin CaMV35S	Southern Blot	1%	Comparison of promoters and optimizing of PIG parameters.	Able et al. 2001 [63]
Immature Embryo	biolistic	pAHC20 pAct1-D	*bar*	Ubi1	*gus*	Actin	Southern Blot	0.18%	Methylation based Silencing of Act1-D	Emani et al. 2002 [64]
Immature Embryo, Mature Embryo, Shoot Tips	biolistic	pAct1-D pAHC25	*bar*, *neo*, *hpt*	Ubi1, Adh1, CaMV35S, ActD	*gus*	Ubi1, Adh1 CaMV35S Act1D	Southern Blot, Gus Assay, PCR	Few events	Tested physical parameters along with different promoters	Tadesse et al. [65]
Immature Embryo	biolistic	pPH1687	*hpt, npt II*	Ubi1	*luc*	Ubi1	Southern Blot	0.09%	Optimizing tissue culture parameters	Raghuwanshi and Birch [66]
Immature Embryo	biolistic	pAHC25 pNOV3604	*bar*, *pmi*	Ubi1	*gus*	Ubi1	PCR, Southern Blot	0.77%	Study the *bar* and *pmi* as selectable marker efficiency.	Grootboom et al. 2010 [67]
Immature Embryo	biolistic	pUKN pGEM-*Ubi-gfp*	*npt II*	Ubi1	*gfp*	Ubi1	PCR, Southern Blot	20.7%	Study the impact of Co bombardment of *npt II* and *gfp,* CuSO_4_	Liu and Godwin [68]

*bar*, Bialaphos resistance; CaMV35S, Cauliflower mosaic virus; *gfp*, Green fluorescent protein; *gus*, β-glucoronidase; *hpt, hph*, Hygromycin phosphotransferase; *luc^+^*, Luciferase; MAS, Mannopine synthase; *npt*, Neomycin phosphotransferase; *pmi*, Phosphomannose isomerase; Zm-Ubi1, Maize Ubiquitin1.

**Table 2:** Different reagents utilized in optimized media composition.

Nutrient Media	Antioxidant	Osmotic Element/Energy Element	Growth Regulators	Vitamins	Anti-Phenolic	Antibiotics to Eliminate *Agrobacterium*	References
MS	ascorbic acid, coconut water	sucrose, glucose	BAP, MES, proline, 2,4-D, IBA, IAA, zeatine, ABA, NAA, thidiazuron	MS vitamin stock (nicotinic acid, pyridoxin HCl, thiamine HCl)	PVP	carbencilline	Zhao et al. [19]
I_6_		glucose	2,4-D	I_6_ based vitamin		cefotaxime	Jeoung et al. [13]
MS	DTT	sucrose, glucose	MES, 2,4-D, proline, asparagine, kinetine, IAA	vitamin B5	PVP, PVPP	cefotaxime, carbencilline, timentin	Carvelho et al. [10]
MS	ascorbic acid	sucrose, glucose	MES, proline, 2,4-D, IBA, IAA	MS vitamin stock (nicotinic acid, pyridoxin HCl, thiamine HCl)	PVP	carbencilline	Gao et al. [20]
MS		sucrose, glucose	kinetin, MES, proline, IAA, 2,4-D,	MS vitamin stock		carbencilline	Howe et al. [22]
MS	AC	sucrose	CH, 2,4-D, IAA, zeatine, proline	MS vitamin B5	AC	carbencilline	Nguyen et al. [25]
MS	ascorbic acid	sucrose, glucose	kinetine, IAA, NAA	MS vitamin stock	PVP	carbencilline	Gurel et al. [6]
MS,N6		sucrose	2,4-D, casamino acid, 6-BA, NAA, sorbitol	MS vitamin stock		cefotaxime	Zhang et al. [58]
I_6_			2,4-D, proline, auxin	MS vitamin stock		cefotaxime	Arulselvi et al. [59]
MS			BAP, 2,4-D	MS vitamin stock		cefotaxime	Jambagi et al. [23]
MS	*L*-cystine	sucrose, glucose	asparagine, kinetine, NAA, 2,4-D, proline, IBA	vitamin B5		cefotaxime, carbencilline	Kumar et al. [38]
MS	*L*-cystine			MS vitamin stock	*L*-cystine	timentin	Pandey et al. [41]
MS	ascorbic acid	sucrose, glucose	proline, 2,4-D, MES, CH, zeatine, IAA, IBA	MS vitamin stock		carbencilline	Wu et al. [40]
MS		sucrose	CH,proline,2,4-D, kinetine, BAP	MS vitamin stock		cefotaxime	Ignacimuthu and Premkumar [60]
MS	ascorbic acid	sucrose, glucose	2,4-D, proline, MES, BAP, IAA, IBA	vitamin B5	PVP	cefotaxime	Do et al. [39]
MS	ascorbic acid coconut water	sucrose, glucose	proline, MES, zeatine, IAA, IBA, aspargine, kinetine	vitamin B5	PVPP	carbencilline, timentin	Mookkan et al. [61]

MS, (Murashige and Skoog, 1962); BAP, 6-Benzylaminopurine; MES, 2-(*N*-morpholino) ethane sulfonic acid; CH, Casein Hydrolysates; 2, 4-D, 2, 4-Dichlorophenoxyacetic acid; IAA, Indole-3-acetic acid; IBA, Indole-3-butyric acid; NAA, Naphthaleneacetic acid; PVP, Polyvinylpyrrolidone; AC, Activated Charcoal; PVPP, Polyvinylpolypyrrolidone; ABA, Abscisic acid; DTT, 1, 4-dithiothreitol.
